# A high-content neuron imaging assay demonstrates inhibition of prion disease-associated neurotoxicity by an anti-prion protein antibody

**DOI:** 10.1038/s41598-022-13455-z

**Published:** 2022-06-09

**Authors:** Madeleine Reilly, Iryna Benilova, Azadeh Khalili-Shirazi, Christian Schmidt, Parvin Ahmed, Daniel Yip, Parmjit S. Jat, John Collinge

**Affiliations:** MRC Prion Unit at UCL, UCL Institute of Prion Diseases, University College London, Courtauld Building, 33 Cleveland Street, London, W1W 7FF UK

**Keywords:** Prions, Cell biology, Cellular imaging

## Abstract

There is an urgent need to develop disease-modifying therapies to treat neurodegenerative diseases which pose increasing challenges to global healthcare systems. Prion diseases, although rare, provide a paradigm to study neurodegenerative dementias as similar disease mechanisms involving propagation and spread of multichain assemblies of misfolded protein (“prion-like” mechanisms) are increasingly recognised in the commoner conditions such as Alzheimer’s disease. However, studies of prion disease pathogenesis in mouse models showed that prion propagation and neurotoxicity can be mechanistically uncoupled and in vitro assays confirmed that highly purified prions are indeed not directly neurotoxic. To aid development of prion disease therapeutics we have therefore developed a cell-based assay for the specific neurotoxicity seen in prion diseases rather than to simply assess inhibition of prion propagation. We applied this assay to examine an anti-prion protein mouse monoclonal antibody (ICSM18) known to potently cure prion-infected cells and to delay onset of prion disease in prion-infected mice. We demonstrate that whilst ICSM18 itself lacks inherent neurotoxicity in this assay, it potently blocks prion disease-associated neurotoxicity.

## Introduction

Prion diseases are progressive and invariably fatal neurodegenerative diseases that involve the misfolding of the cellular prion protein (PrP^C^) into prions—self-propagating fibrillary assemblies of disease-associated PrP—that spread throughout the brain^1^. Hitherto, attempts to develop effective therapeutics for prion diseases have focused on measuring prion propagation in cell models or incubation periods in prion-infected rodents. However, studies of the kinetics of prion propagation in mice revealed that prion propagation and neurotoxicity can be uncoupled^2,3^. Following an initial phase of exponential prion propagation, not rate limited by PrP^C^ expression level, infective titre reaches a plateau level. The length of this second, plateau, phase to clinical onset is inversely proportional to PrP^C^ expression level^2^. During this second phase there is accumulation of protease-sensitive, disease-related forms of PrP at a rate linearly proportional to PrP^C^ expression level and neuropathology becomes established^3^. We hypothesised that alternate, toxic, PrP species (designated PrP^L^) are generated and that clinical onset occurs when these reach a local neurotoxic threshold^2–4^. This model proposes that infectious prions are themselves not toxic but rather that a pathway switch to production of PrP^L^ once a plateau level of prion infectivity is reached accounts for the synaptotoxicity and neurodegeneration that ultimately leads to clinical onset. Such a model is also consistent with the existence of subclinical prion infections where animals live a normal lifespan despite harbouring high brain prion titres^5,6^. Subsequent studies demonstrated that highly purified prions are indeed not directly neurotoxic^7^. Such interplay of propagating and toxic PrP assemblies, and how this leads to neurodegeneration, may also be of importance to understanding the commoner neurodegenerative diseases involving pathogenic fibrillary assemblies of other host proteins, notably Alzheimer’s disease (AD)^1,8,9^.

While the precise mechanism of neurotoxicity in prion neurodegeneration remains unclear, PrP^C^ is the obligate substrate for production of such toxic PrP species as well as prion propagation. In addition, there is also evidence that PrP^C^ acts as a receptor for toxic species not only in prion disease, where susceptibility critically depends on PrP^C^ expression^10–12^, but also in AD and other neurodegenerative conditions^13,14^. Depletion of neuronal PrP^C^ expression during established neuroinvasive prion infection prevents clinical disease and development of neuropathology despite continued production of prions by non-neuronal cells^15,16^. Furthermore, acute toxicity of prion-infected brain samples in cell culture depends on neuronal PrP^C^ expression^17^.

Putative therapeutic approaches that target PrP^C^ might be more effective at blocking production of disease-related PrP species that accumulate during the plateau phase of prion infection than prion propagation itself since the former is rate limited by PrP^C^ concentration and the latter is not. For these reasons, we recently developed a high-content cellular assay that allowed accurate measurement of prion disease-associated toxicity in brain samples^7^ rather than prion propagation and have now extended this assay to permit testing of candidate therapeutics that target neurotoxicity specifically rather than prion propagation.

Monoclonal antibodies targeting PrP have been proposed as therapeutics for prion disease and shown to be able to cure prion-infected cells in vitro and significantly delay clinical onset in prion-infected mice. However, a number of studies have reported significant neurotoxicity with various anti-PrP antibodies raising concerns about human therapeutic use of anti-PrP antibodies^18–20^. These effects appear variable and either highly antibody specific^18^ or occurring at antibody concentrations far exceeding efficacious therapeutic dose ranges^20^. Variability in the antibodies themselves and the use of diverse methods and cell models to monitor neurotoxicity has led to a complex published data set regarding the relative-safety of immunotherapeutic approaches to treating prion disease in humans^18–21^.

Here we have applied our cellular assay of prion disease-related neurotoxicity to study the mouse monoclonal antibody, ICSM18, which recognises amino acid residues 143–153 of murine PrP^22^. ICSM18 has been previously shown to cure prion infection in prion-infected cells^23,24^ and to delay clinical onset in prion-infected mice^25^. ICSM18 has been fully humanized as a candidate human therapeutic^19,26^. Potency of antibody curing of prion-infected cells correlates with binding affinity for PrP^C^ rather than disease-associated PrP, suggesting cure by preventing PrP^C^ recruitment into pathological fibrillary assemblies^22^. The development of this high content cellular assay for prion disease-related neurotoxicity has allowed us to both evaluate the potential inherent toxicity of ICSM18 and also investigate its therapeutic potential for abrogating prion disease-associated neurotoxicity. We report that ICSM18 can potently antagonise prion-disease related neurotoxicity in the absence of any measurable antibody-mediated toxic effects on neuronal health in our model.

## Results

### A high-content cellular assay for prion disease-associated neurotoxicity

Recently we developed a neuron-based imaging assay of prion disease-associated neurotoxicity and reported that while prion-infected brain samples had high levels of neurotoxicity, highly purified infectious prions did not^7^. We have now extended the performance of the previously established method to demonstrate high signal-to-noise windows between prion-infected and uninfected brain homogenate samples through a multiple-logarithmic dynamic range, permitting the investigation of potential anti-neurotoxic therapeutics.

**Figure 1:**
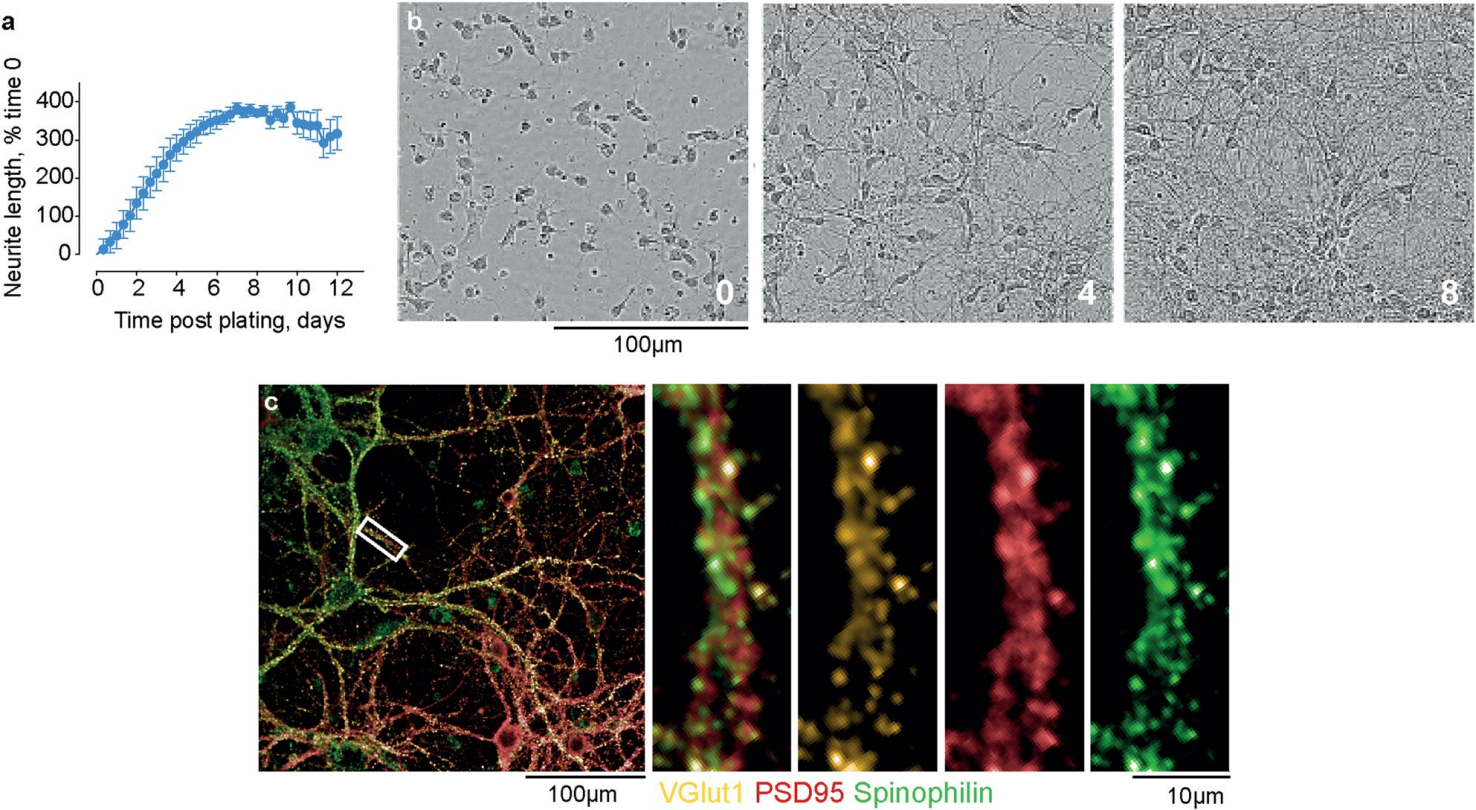
Characterisation of neuronal cell culture model. (**a**,**b**) The neurite length of hippocampal neurons was calculated using the NeuroTrack analysis add-on for an IncuCyte S3 live cell imager. Neurite length was plotted over time post-plating: shown as the mean ± SEM of 3 images from 4 wells per condition and in images at time 0, 4 and 8 days. (**c**) Hippocampal neurons were maintained in culture and fixed at 10 days post-plating. The left hand image is a merge of pre- (VGlut1, orange) and post- (PSD-95, red) synaptic markers co-stained with the dendritic spine marker, spinophilin (green). The view within the white box was magnified and shown as a merge of all channels, and as individual channels**.**

Primary hippocampal neurons were prepared and maintained in culture as described previously^7^. By day 10 in vitro*,* neurite lengthening is complete (Fig. 1a,b) and neurons present mature synapses (as indicated by the co-localisation of pre- and post-synaptic markers) (Fig. 1c); there was also a clear localisation of synapses to dendritic spines (Fig. 1c). Cells were plated at optimal densities to limit neuron–neuron overlap (Fig. S1), thereby facilitating accurate segmentation of regions of interest and objects during subsequent image analysis. The selected neuronal markers for high-content imaging were NeuN, MAP2 and spinophilin^27,28^, which enabled neuron counting and nuclear morphology measurements (Fig. S2), neurite tree profiling (Fig. S3), the assessment of neurite damage (Fig. S4) and dendritic spine/synaptic measurements (Fig. S5) in tandem. Fixing, staining and imaging protocols were optimised for accurate boundary and object identification by Acapella™ image analysis algorithms within Columbus, a cloud image repository and analysis interface (Figs. S2–S5).

### Prion disease-associated neurotoxicity results in multiple phenotypic changes in neurons

**Figure 2:**
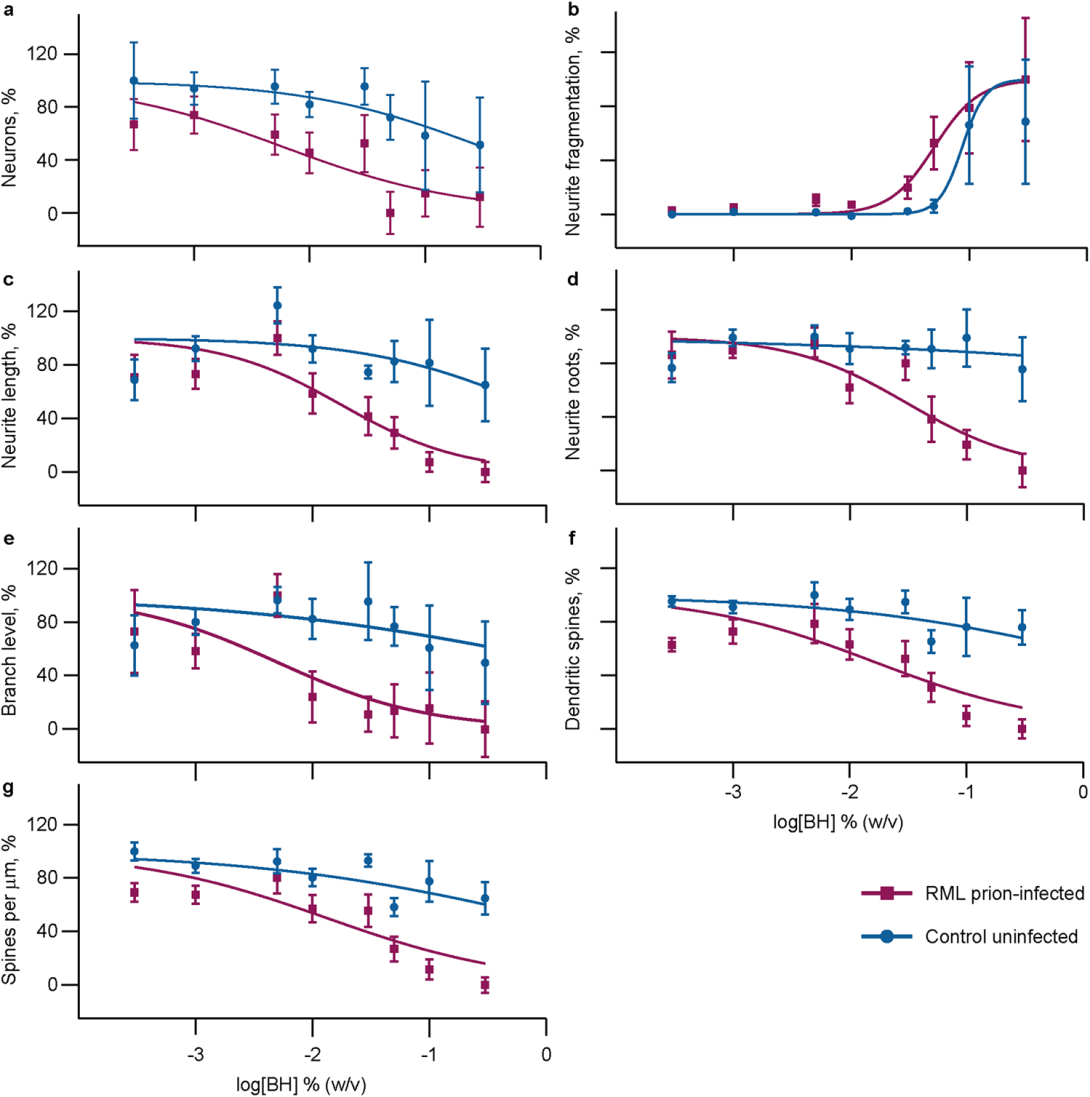
Prion-infected brain homogenate neurotoxicity. 10–13 day old mixed cortical–hippocampal neurons were treated with serial dilutions of control uninfected brain homogenate and RML prion-infected brain homogenate for 72 h before fixation, staining and imaging. Images were run through analysis scripts that counted neurons (**a**), calculated neurite fragmentation per neuron (**b**), neurite length (**c**), root number (**d**) and branch level (**e**), counted dendritic spines (**f**) and calculated their density along dendrites (**g**). The baseline (media-only) was removed on a per plate basis from raw data—each independent test was the average of 3–4 wells per condition with 15 images taken in each well. Data were normalised between 0 and 100% and shown as mean ± SEM of *n* = 5–20 independent tests per concentration; data were transformed to log10 before non-linear regression using Y = Bottom + (Top − Bottom)/$({1} + {1}0^{{(({\text{LogEC}}_{{{5}0}} \, - \,{\text{X}})\, \times \,{\text{HillSlope}})}} )$ and an extra sum-of-squares *F* test tested whether best-fit values of logEC_50_ differed between the two data sets.

Neurons were treated with serial dilutions of RML prion-infected and control uninfected brain homogenates, done over multiple independent tests per concentration to create standard toxicity curves. This facilitated estimating brain homogenate-induced effect sizes and validated the specificity, reproducibility, limits of detection, working range and precision of the assay. RML prion-infected brain homogenate in comparison to control reduced the neuron population (logEC_50_ = − 2.4 vs. − 0.7; P < 0.0001) (Fig. 2a). The extent to which neurites fragmented was higher on average for RML prion-infected brain homogenate-treated neurons compared to uninfected control (logEC_50_ = − 1.3 vs. − 1.1; P = 0.0472) (Fig. 2b), as was the extent of neurite shortening (logEC_50_ = − 1.8 vs. − 0.4; P = 0.0002) (Fig. 2c), neurite root reduction (logEC_50_ = − 1.2 vs. − 0.2; P < 0.0001) (Fig. 2d) and branch degeneration (logEC_50_ = − 2.3 vs. − 0.2; P = 0.0016) (Fig. 2e). RML prion-infected brain homogenate also induced significantly more dendritic spine loss than control in terms of total spines (logEC_50_ = − 1.8 vs. 0.4; P < 0.0001) and spine density (logEC_50_ = − 1.9 vs. 0.03; P < 0.0001) (Fig. 2f,g).

**Figure 3:**
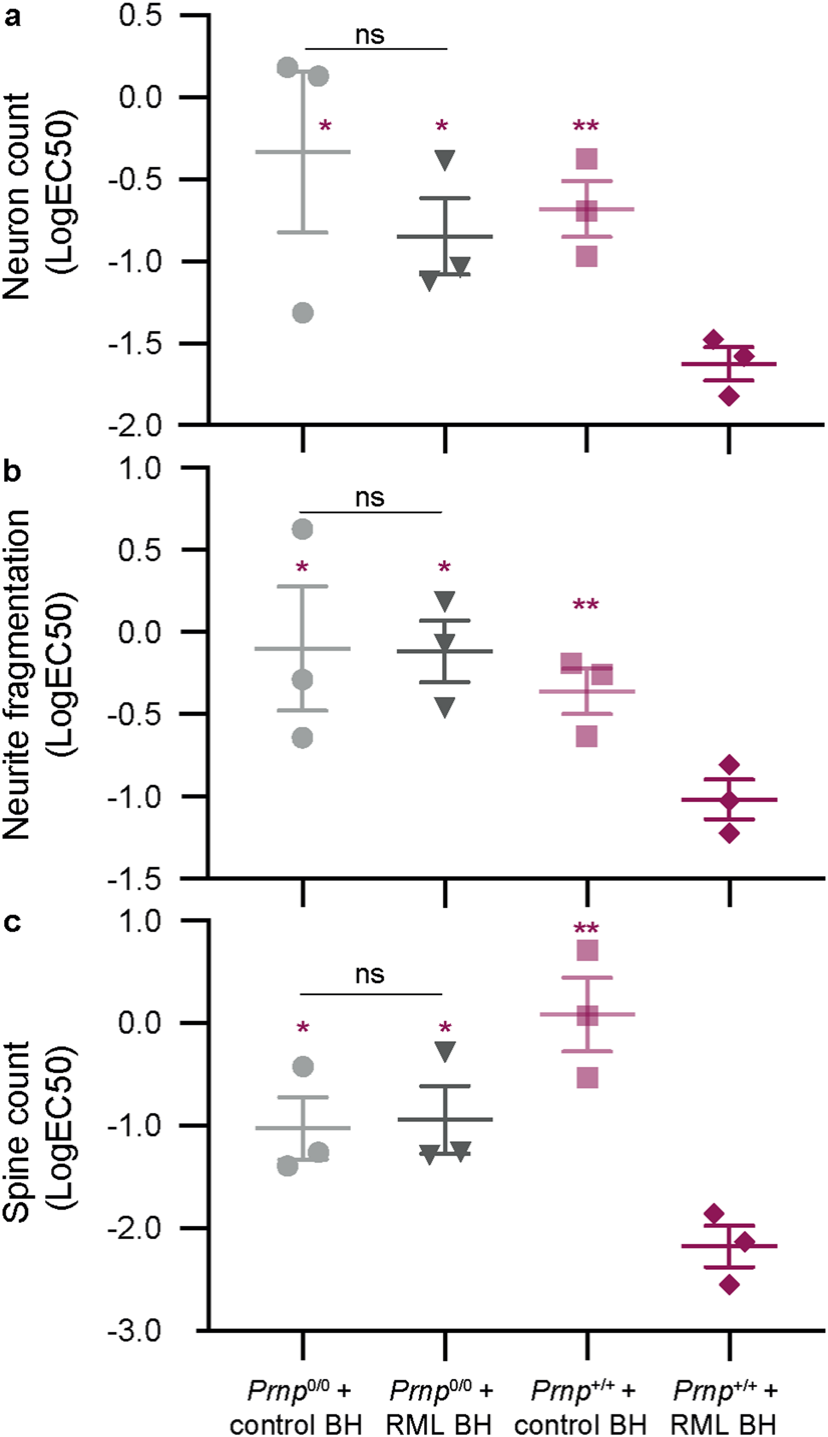
Prion-infected brain homogenate toxicity depends on PrP^C^. PrP^C^ wild-type (*Prnp*^+*/*+^) and knock-out (*Prnp*^0/0^) mixed cortical–hippocampal neurons were maintained in culture for 10 days before a 72 h treatment with serially diluted RML prion-infected and control uninfected brain homogenate (BH) or with medium only. Cells were then fixed and stained with the standard panel of antibodies: anti-NeuN, MAP2 and spinophilin. Images were analysed with standard Columbus image analysis scripts. A logEC_50_ value was computed by Y = Bottom + (Top − Bottom)/${1} + {1}0^{{(({\text{LogEC}}_{{{5}0}} \, - \,{\text{X}})\, \times \,{\text{HillSlope}})}}$ for the neuron count (**a**), neurite fragmentation (**b**) and dendritic spine count (**c**) analyses; data are shown as the mean ± SEM of n = 3 independent tests. Statistics correspond to independent two-tailed *t* tests between the logEC_50_ values of RML prion-infected brain homogenate in *Prnp*^+*/*+^ cells vs. all other conditions (red) or RML prion-infected vs. control uninfected brain homogenate in *Prnp*^*0/0*^ cells (black); *P < 0.05, **P < 0.01, *ns* not significant.

The neurotoxicity of RML prion-infected brain homogenate also depended on the neuronal expression of PrP^C^ (Fig. 3). Overall, RML prion-infected brain homogenate could be discerned from that of uninfected control at multiple levels of neurotoxicity and through a wide dynamic range; the majority of analyses found significant differences between infected and uninfected toxicity at concentrations over 0.001 and under 0.1% (w/v). The average logEC_50_ value for RML-infected brain homogenate was − 1.8 [~ 0.01% (w/v)] whilst control was − 0.3.

### ICSM18 is not inherently toxic to hippocampal neurons

**Figure 4:**
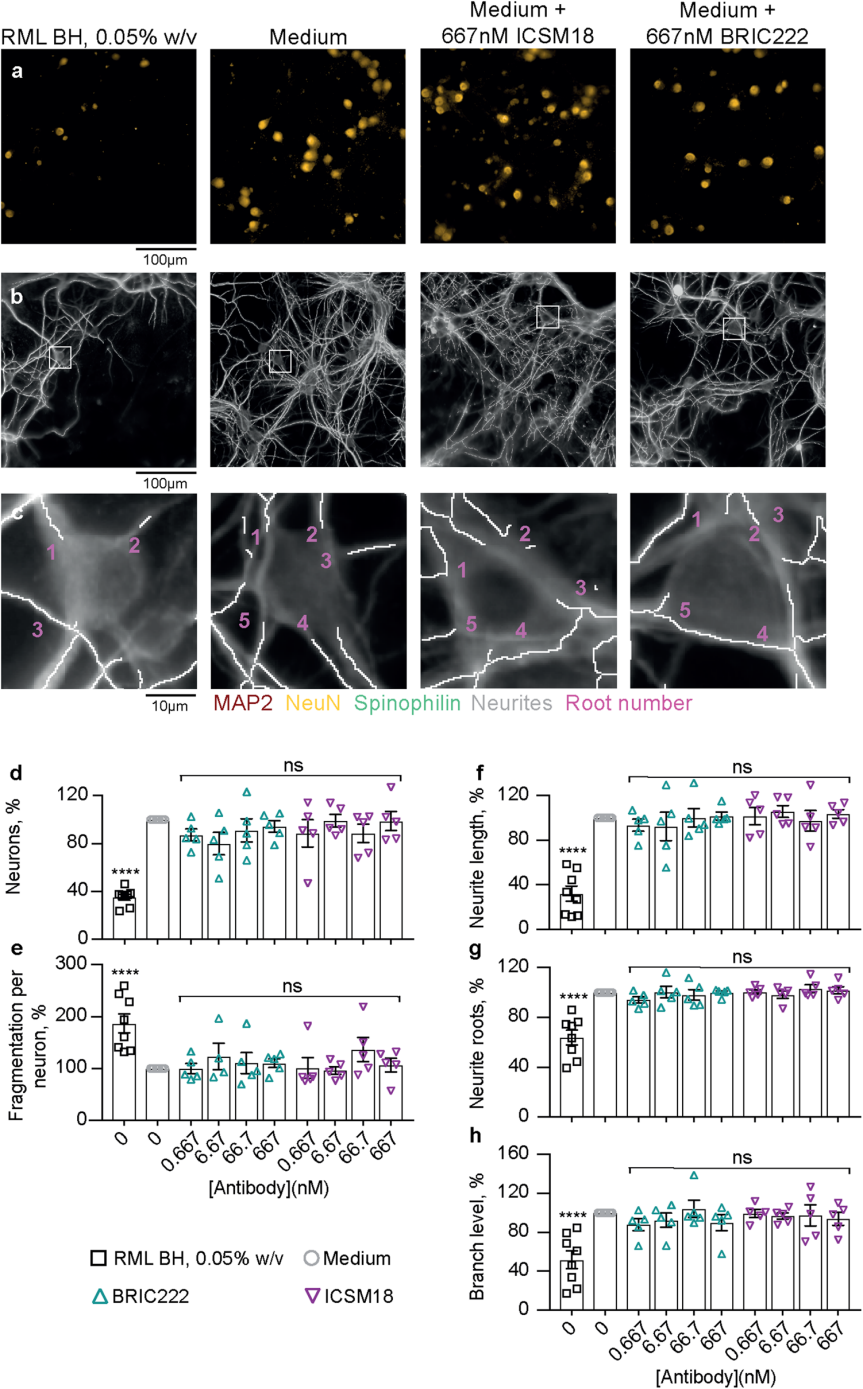
ICSM18 is not inherently neurotoxic. Hippocampal neurons were treated with ICSM18 antibody and its isotype control, BRIC222, with and a positive control [0.05% w/v RML-infected brain homogenate (BH)] and negative (media only) control for 72 h. (**a**) NeuN (orange) stain and (**b**) and MAP2 (grey) overlaid with the neurite trace (white)**.** (**c**) Magnified view of the white box in (**b**) showing a representative neuron nucleus and neurite root count. Images were run through analysis scripts that counted (**d**) neurons and (**e**) neurite fragmentation and calculated (**f**) neurite length, (**g**) root density and (**h**) branch level. Data are the percentage difference from the negative control and shown as mean ± SEM of *n* = 5 independent tests; one-way ANOVA with Dunnett’s multiple comparisons; ****P < 0.0001; *ns* not significant.

**Figure 5:**
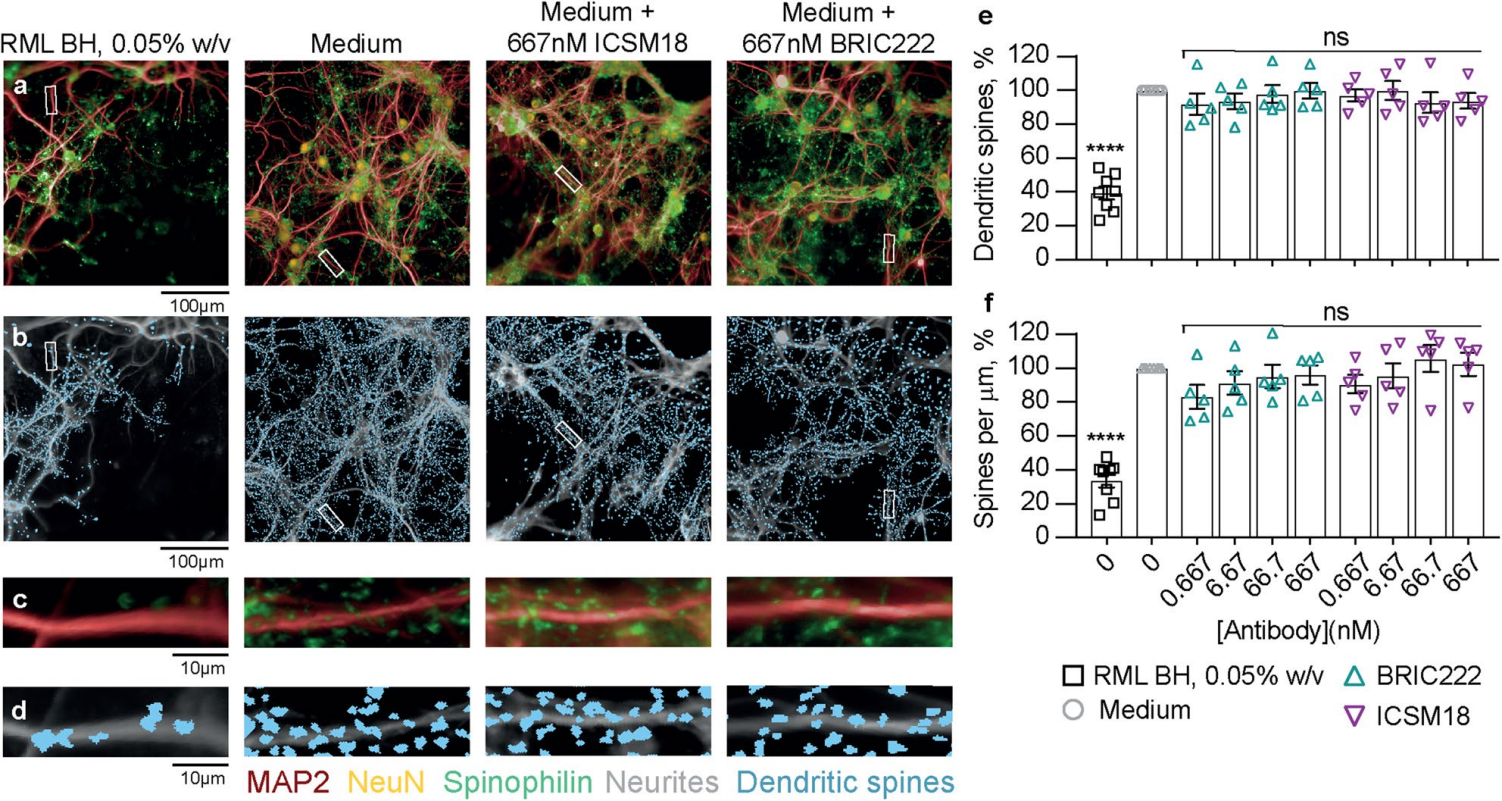
ICSM18 is not inherently synaptotoxic. Hippocampal neurons were treated with ICSM18 antibody and its isotype control, BRIC222, with and a positive control [0.05% w/v RML-infected brain homogenate (BH)] and negative (media only) control for 72 h. (**a**) Representative high-throughput images of cells stained with MAP2, spinophilin and NeuN to visualise dendrites, dendritic spines and neuronal nuclei respectively. (**b**) Dendritic spine image analysis mask (blue). (**c**,**d**) Magnified views of the white rectangles in (**a**) and (**b**). Images were run through analysis scripts that counted (**e**) dendritic spines and calculated (**f**) spine density; results are the percentage difference from the negative control and shown as mean ± SEM of *n* = 5 independent tests; one-way ANOVA with Dunnett’s multiple comparisons; ****P < 0.0001; *ns* not significant.

Hippocampal neurons were treated with ICSM18 and its IgG isotype control, BRIC222, through a range of concentrations (0.67–667 nM) for 72 h. The maximal concentration of ICSM18 tested was 100 times higher than in a study reporting ICSM18-induced dendritic beading^21^. Neuron population numbers (Fig. 4a) and neurite architecture (Fig. 4b,c) appeared to be unaffected by both ICSM18 and BRIC222 antibody treatments. Image analysis showed that the total number of neurons did not change with increasing concentrations of antibody (Fig. 4d). Neurite fragmentation, length, root density and branch level were all similarly unaffected (Fig. 4e–h). There was also no evidence of ICSM18 or BRIC222-mediated synapse toxicity (Fig. 5a–d), quantified in terms of total dendritic spine counts (Fig. 5e) and spine density along dendritic processes (Fig. 5f). A 0.05% (w/v) RML prion-infected brain sample, in contrast, induced clear neurotoxicity in all assay outputs (Figs. 4, 5). The biological activity of ICSM18 was verified by a prion infectivity curing assay in chronically prion-infected iPK-1 mouse neuroblastoma cells (EC_50_ = 1.6 nM) (Fig. S6a)^23,24^. In subsequent experiments, ICSM18 was tested for its potency in a cell curing assay in tandem with toxicity. Whilst ICSM18 was highly potent in curing a prion infection (EC_50_ = 0.8 nM) (Fig. S6b), no cellular toxicity was observed. In summary, we were unable to detect any antibody-mediated neurotoxicity, up to and including 667 nM ICSM18, a concentration far in excess of its cell curing EC_50_ (Figs. 4, 5).

### ICSM18 blocks prion disease-associated neurotoxicity

To test whether ICSM18 could antagonise prion disease-associated neurotoxicity, hippocampal neurons were pre-treated with ICSM18 and BRIC222 antibodies for 1.5 h at a range of concentrations between 0.67 and 667 nM. RML prion-infected or control uninfected brain homogenates were then added to a final concentration of 0.01% (w/v). This concentration was selected to maximise the chance of observing ICSM18 activity and was also equivalent to the average logEC_50_ (− 1.95) of RML-infected brain homogenate neurotoxicity (Fig. 2).

**Figure 6:**
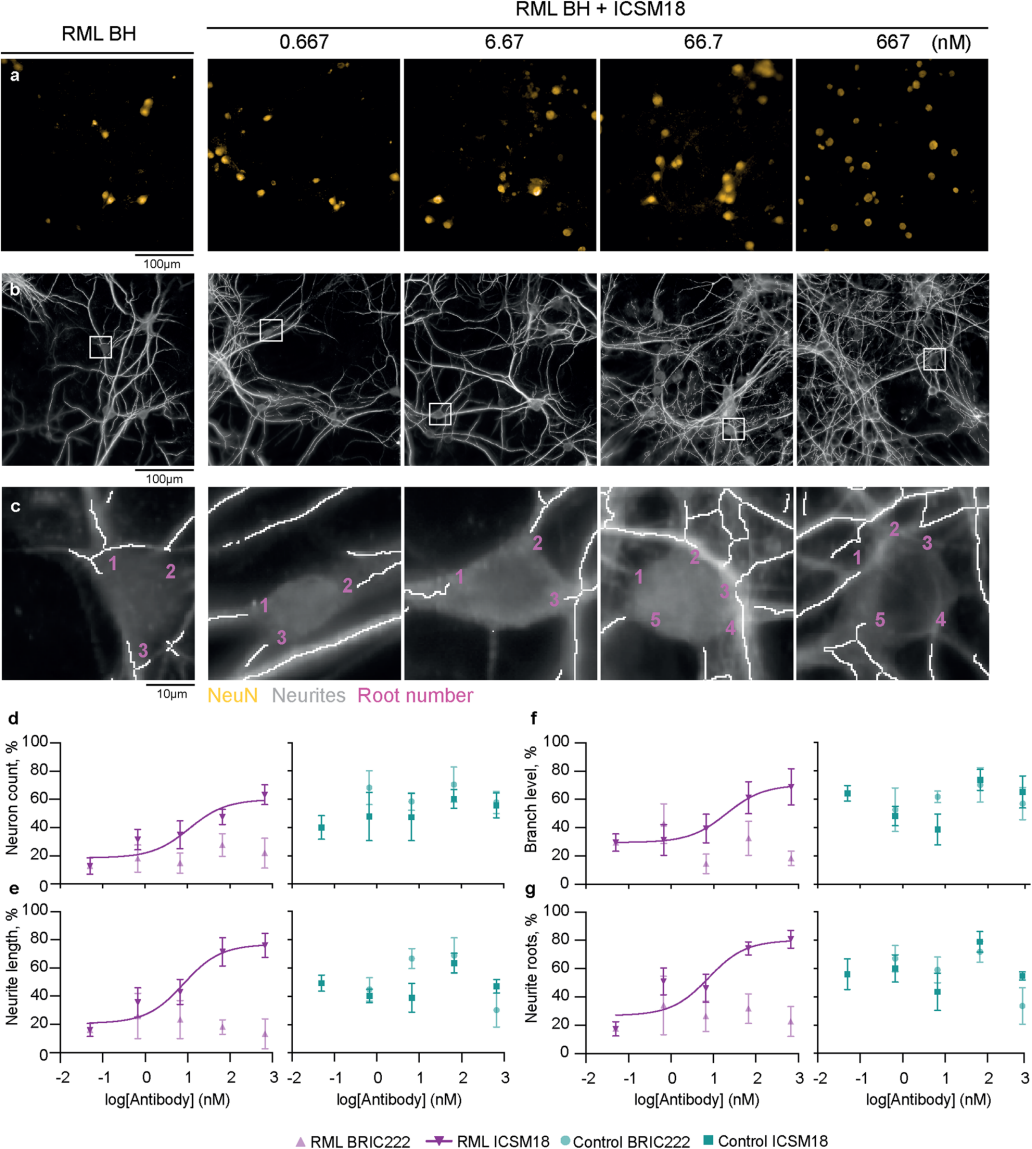
ICSM18 inhibits prion disease-associated neurotoxicity**.** Hippocampal neurons were treated with ICSM18 antibody and its isotype control, BRIC222 for 1.5 h before control uninfected or RML-infected brain homogenate (BH) was added to a final concentration of 0.01% w/v. Brain homogenate and antibodies were then co-incubated for a further 72 h before fixation and staining. (**a**) NeuN (orange) stain and (**b**) MAP2 (grey) overlaid with the neurite trace (white). (**c**) Magnified view of the white box in (**b**), showing a representative neuron nucleus and neurite root count. Images were run through analysis algorithms that counted (**d**) neurons and calculated (**e**) neurite length (**f**) branch level and (**g**) neuron root density. The baseline was removed on a per plate basis and then normalised between 0 and 100% on a batch basis in order to run non-linear regression and shown as mean ± SEM of *n* = 5 independent tests; non-linear regression analysis used the equation Y = 100/${1} + {1}0^{{(({\text{LogEC}}_{{{5}0}} \, - \,{\text{X}})\, \times \,{\text{HillSlope}})}}$ and assumed antibody-negative RML treatment group as 0.05 nM.

**Figure 7:**
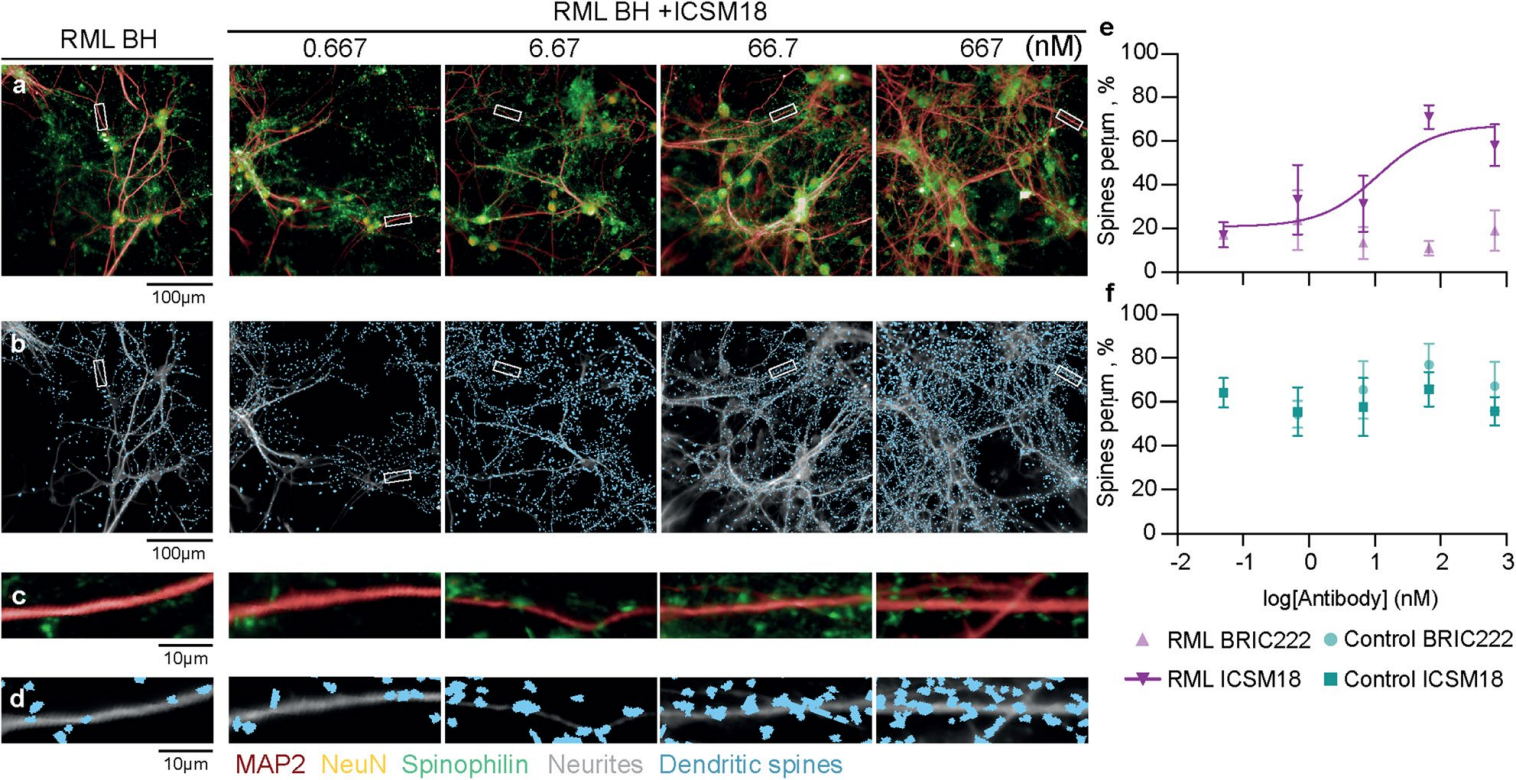
ICSM18 inhibits prion-induced synaptotoxicity. Hippocampal neurons were treated with ICSM18 antibody and its isotype control, BRIC222 for 1.5 h before control or RML-infected brain homogenate (BH) was added to a final concentration of 0.01% w/v. BH and antibodies were then co-incubated for a further 72 h before fixation and staining. (**a**) Representative high-throughput images of cells stained with MAP2, spinophilin and NeuN to visualise dendrites, dendritic spines and neuronal nuclei respectively. (**b**) Dendritic spine image analysis mask (blue). (**c**,**d**) The white rectangles in (**a**) and (**b**) correspond to magnified views in (**c**) and (**d**) respectively. Dendritic spine density analysis for cells incubated with ICSM18 and BRIC222 before (**e**) RML-infected or (**f**) control brain homogenate treatment; The baseline was removed on a per plate basis and then normalised between 0 and 100% on a batch basis in order to run non-linear regression and shown as mean ± SEM; *n* = 5 independent tests; non-linear regression analysis used the equation Y = 100/${1} + {1}0^{{(({\text{LogEC}}_{{{5}0}} \, - \,{\text{X}})\, \times \,{\text{HillSlope}})}}$ and assumed antibody-negative RML treatment group as 0.05 nM.

ICSM18 antagonised the toxicity of RML prion-infected brain homogenate treatment in a dose-dependent manner, with respect to both neuron population numbers (Fig. 6a) and neurite architecture (Fig. 6b,c). ICSM18 prevented RML-infected brain homogenate-mediated neuron loss (EC_50_ = 11.0 nM) (Fig. 6d), neurite shortening (EC_50_ = 8.0 nM) (Fig. 6e), branch degeneration (EC_50_ = 20.0 nM) (Fig. 6f) and reduction in neuron root density (EC_50_ = 7.7 nM) (Fig. 6g). BRIC222, conversely, had no effect on the toxicity of RML-infected brain homogenate, irrespective of the concentration tested (Fig. 6d–g). Furthermore, both ICSM18 and BRIC222 did not affect the toxicity of uninfected brain homogenate (Fig. 6d–g). ICSM18 also antagonised RML prion-infected brain tissue-mediated synapse toxicity (Fig. 7a–d), quantified in terms of dendritic spine density (EC_50_ = 11.1 nM) (Fig. 7e), whereas BRIC222 did not. Neither ICSM18 nor BRIC222 affected the synaptotoxicity of uninfected brain homogenate (Fig. 7f).

## Discussion

Recently we reported a high-content cell imaging assay that facilitated the measurement of acute prion disease-associated neurotoxicity^7^. We have extended this assay to determine multiple neurotoxic phenotypes in neurons after treatment with prion-infected brain homogenates through a wide concentration range, establishing sample effect sizes, assay specificity, reproducibility, precision, its limits of detection and working range. Whilst uninfected brain homogenate exhibited low levels of non-specific neurotoxicity, prion-infected brain homogenate induced much higher levels of toxicity, which was also cell PrP^C^ expression-dependent. Our results (^7^ and this study) are consistent with previously reported assays using primary neurons^17^ and organotypic slice cultures^29,30^. The novel assay reported here also detected acute toxicity of prion-infected brain homogenate, but over a wider dynamic range and with high-throughput imaging and image analysis, which will ultimately facilitate rapid assessment of anti-neurotoxic drugs and expedite the isolation and purification of the toxicity-inducing species in prion infected brains. Importantly, we demonstrate that ICSM18, a mouse monoclonal antibody that has been humanised for human therapeutic use^26^, is not inherently neurotoxic or synaptotoxic and indeed that it can potently inhibit prion disease-related neurotoxicity and synaptotoxicity.

Initially anti-PrP antibodies were devised as a therapeutic strategy for blocking prion propagation^25^. However, more recent evidence suggested that antibodies binding PrP^C^ should also inhibit production of toxic PrP assemblies which appear distinct from infectious prions themselves^2,3,7^. However, the safety of a passive immunotherapy strategy has been questioned with reports of significant toxicity of anti-PrP monoclonal antibodies in various in vitro models and following direct injection into mouse brain^18–21^. We were unable to reproduce the original finding of direct neurotoxicity on injection of anti-PrP antibodies into mouse hippocampus and other studies have challenged the specificity and relevance of in vitro studies to human therapeutics^19,31,32^. Studies on ICSM18’s inherent toxicity to cells were therefore undertaken to clarify contradictory reports of potential ICSM18 neurotoxicity. In a robust examination of potential ICSM18 toxicity in primary hippocampal neurons reported here, biologically active ICSM18 was found to elicit no neurotoxic effects over a wide concentration range. This included concentrations 100-fold greater than a concentration previously reported by others to induce dendritic beading in vitro^21^. The morphological appearance of dendritic beading^21^ is analogous to the neurite fragmentation analysis reported here, however, dendritic beading induced by ICSM18 was reported only in Tga20 hippocampal neurons overexpressing PrP^C^ eight times wild-type levels.

Further, we now show that ICSM18 blocks prion disease-associated toxicity in vitro suggesting that synaptotoxicity and neurodegeneration—not simply prion propagation -can be directly targeted in vivo. The antagonistic effect of ICSM18 on prion disease-associated toxicity likely relied on an interaction with cell surface PrP^C^. PrP^C^ was required for the toxicity induced by prion-infected brain homogenate and ICSM18, known to bind native PrP^C^ rather than disease-related isoforms, antagonised this toxicity. Furthermore, the neuronal cells were not actively prion-infected^7^ meaning, therefore, that toxic activity resided in a component of the prion-infected brain samples. As we now know that infectious prions themselves are not inherently neurotoxic^7^, toxicity in our neuronal assay was likely caused by alternative forms of disease-associated PrP within brain samples. This study has not attempted to characterise the entities responsible for toxicity or examine the mechanism of toxicity specifically. However, this assay for specific prion disease-related neurotoxicity should facilitate studies to isolate and characterise such toxic species and determine their mechanisms of action.

It is now clear that ICSM18 can block the activity of prion disease-related neurotoxic species which accumulate following plateauing of prion propagation in mice. It will be important to address whether ICSM18 can antagonise toxicity in clinically affected mice in addition to its effect on the asymptomatic incubation period as this may be relevant to human therapeutic use of anti-PrP monoclonal antibodies.

## Materials and methods

### Study design

Estimates for brain homogenate-induced neurotoxic effect sizes were validated for the high-content toxicity assay by generation of standard curves for prion-infected and uninfected brain tissue. Here, sample sizes were purposefully large in order to validate the specificity, limits of detection, working range and precision and accuracy of the assay. Power calculations were then made to maximise the power of detecting effects at certain concentrations for neurotoxicity assays that compared brain homogenate toxicity in genetically different cells, measured inherent antibody toxicity and the effect of antibodies on brain homogenate toxicity. Power calculations were also used for infectivity curing assay. Quality control criteria were established prospectively and all data were collected at a predetermined endpoint. Technical replicates were defined as the number of wells in each multi-well plate. For image analysis, each technical replicate was the combined value from 15 images per well. Biological replicates were defined as averaged technical replicates in independent cell cultures (units of investigation). The objective of the research was to establish a powerful assay of prion-infected brain homogenate-induced toxicity for pre-clinical testing of candidate therapeutics for prion disease. The study was a controlled laboratory set-up, where diluted brain homogenate and/or antibody was treated to primary neuronal cells for toxicity analysis or cell lines in culture for infectivity and toxicity analysis. Toxicity in infected cell lines was detected by measuring luminescence using a plate reader. Toxicity in neurons was detected by high-content imaging and image analysis. Infectivity was detected by measuring relative intensity on a plate scanner. Plate layouts were defined before treatment and used matched case–control spacing. The experimenters were not blinded to initial sample allocation, but all image analysis was performed in automated unbiased batches.

### Safety and ethical declaration

All experimental procedures involving RML prions were carried out in microbiological containment level 2 facilities with strict adherence to safety protocols and guidelines. All procedures involving animals were performed under approval and license granted by the UK Home Office (Animals Act 1986; Project License number 70/9022) and conformed to institutional guidelines and are reported in accordance with the Animal Research: Reporting of In Vivo Experiments (ARRIVE) guidelines.

### Prion inoculation and brain homogenate preparation

Mouse-adapted RML prions used in this study were originally supplied by Charles Weissmann, Zurich Institute for Molecular Biology, in 1995. A batch of 200 brains from terminally-infected intracerebrally-inoculated CD1 mice (Hsd:ICR (CD1); Harlan), which were culled at confirmed scrapie sickness by exposure to CO_2_, were homogenised in Dulbecco's phosphate buffered saline lacking Ca^2+^ or Mg^2+^ ions (D-PBS; Invitrogen) using tissue grinders as described previously^33^ to produce an approximately 1 L pool of 10% (w/v) RML brain homogenate (designated I13100). Control 10% (w/v) brain homogenate was prepared in D-PBS from the brains of uninfected CD1. All brain homogenates were stored as aliquots at − 70 °C. Dilutions for analysis were prepared from the initial 10% (w/v) stock.

### Antibodies

The biological activity of ICSM18 has previously been described by the curing of RML-prion infected cells^23,24^. Chronically RML prion-infected iPK1 (subclone of the murine neuroblastoma N2a cell line) cells were incubated for 4 days either in the presence of 5 pM to 300 nM (curing of infection experiment) or 66.7 pM to 66.7 nM (curing of infection experiment in tandem with cytotoxicity testing) of IgG purified hybridoma supernatants of mouse monoclonal antibodies ICSM18 (D-Gen Ltd., London) and isotype control, BRIC222 (IBGRL, Bristol). Additionally, positive (FeT1239, 10 µM (curing of infection experiment) or 5000 Da dextran sulphate, 2 µM (curing of infection experiment in tandem with cytotoxicity testing) and negative (cell-only) controls were included on each plate. For PrP scrapie (PrP^Sc^) detection by dot blot, cells were lysed and transferred to a nitrocellulose membrane, proteinase K-treated and denatured with guanidine thiocyanate. PrP^Sc^ was detected with ICSM18 followed by IRDye 800CW goat anti-mouse IgG (LI-COR^®^ Biosciences), measuring the relative intensity using Odyssey software. The same batch of ICSM18 was used for all assays. If cells were analysed for cytotoxicity in tandem with infectivity, they were analysed with CellTiter-Glo^®^ Luminescent Cell Viability Assay (Promega) using the manufacturers protocol, and luminescence read on a Tecan F200 plate-reader.

### Primary neuronal culture

Cultures were derived from brains of inbred FVB/N (*Prnp*^1/1^; wild-type PrP^C^ expression level) or *Prnp* null^34^ (backcrossed to FVB/N mice; *Prnp*^o/o^; no PrP^C^ expression) mice. Mice were culled by exposure to CO2, before extracting whole brains from pups. Hippocampi and/or cortices of E17 mouse brains were dissected in calcium and magnesium-free Hank’s Balanced Salt Solution (HBSS) supplemented with 2 mM l-glutamine, 10 mM 4-(2-hydroxyethyl)-1-piperazineethanesulfonic acid (HEPES) and 100 U/ml penicillin–streptomycin, dissociated using 0.25% (w/v) trypsin and 1000 U benzonase (VWR), triturated mechanically and live cells counted using Neubauer haemocytometer. Cells were plated in Dulbecco's Modified Eagle's Medium (DMEM) supplemented with 10% (v/v) heat-inactivated horse serum (26050-88, Invitrogen) at 300 cells/mm^2^ to poly-l-lysine coated 96 well plates (655936, Greiner). At 1 h post-plating, DMEM medium was aspirated and exchanged for Neurobasal™ medium (21103049, Thermofisher Scientific) supplemented with 0.25% (v/v) Glutamax™ (35050061, Thermofisher Scientific) and 2% (v/v) Gibco™ B27 supplement (17504044, Thermofisher Scientific) and incubated at 37 °C, 5% CO_2_.

### Live-cell imaging

To characterise the neuronal cell culture in terms of neurite lengthening, images were acquired with an IncuCyte S3, Sartorius. The imaging platform was kept inside a tissue culture incubator at 37 °C and 5% CO_2_. 3 images were taken in each well with a 20 × objective lens in phase contrast from the same well coordinates every 8 h, from cell plating for 12 days. Images taken on IncuCyte were analysed with the NeuroTrack module, IncuCyte S3 v.2018B.

### Immunofluorescence

Cells were fixed in 3.6% phosphate-buffered formaldehyde for 15 min, permeablised in 0.3% TritonX100 for 5 min and blocked in D-PBS containing 5% (v/v) horse serum and 3% (v/v) bovine serum albumin. Primary antibodies were then added and incubated at 4 °C overnight. Antibodies were MAP2 (1:500, M13-1500, Thermofisher Scientific or 1:500, ab5392, Abcam), spinophilin (1:800, 06-825, Millipore), V-Glut1 (135304, Synaptic Systems) and/or PSD-95 (1:500, ab18258, Abcam). Cells were washed with D-PBS and blocked with 5% (v/v) goat serum in D-PBS for 45 min, after which cells were incubated with goat-secondary antibodies coupled to Alexa Fluor^®^ dyes (at a final concentration of 1:1000, Thermofisher Scientific) for 1.5 h at 22 °C. Cells were then washed with D-PBS. If pre-conjugated antibody, NeuN Alexa Fluor^®^ 555 (1:500, MAB377A5, Millipore), was used cells were incubated for a further 1 h at 22 °C, before being washed. If nuclei were then stained, cells were incubated with DAPI (1:2000, D1306, Molecular Probes) for a further 20 min and then washed. 100 µl D-PBS was added to all stained wells of each 96-well plate and subsequently sealed and stored in the dark at 4 °C.

### Image acquisition for high-content neurotoxicity assay

Images of fixed cells were taken on an Opera Phenix, Perkin Elmer high-throughput microscope with a 40 × objective water immersion lens using Harmony, Perkin Elmer software. 15 fields of view were captured in each well. The laser power and exposure times were constant for each channel over each batch of images acquired. The height of the objective was optimised on a per plate basis. Flatfield correction profiles were estimated at imaging.

### Image analysis for Opera Phenix high-content imaging

All images collected on the Opera Phenix were run through standard Acapella™ analysis scripts in Columbus, Perkin Elmer. Flatfield correction was applied as the starting point for all analyses.

Neuron counts were made by segmenting NeuN-positive nuclei using the ‘Find nuclei’ function. Nuclear morphological measurements [area (µm^2^) and roundness (perimeter form factor (Ff) calculated from a function of perimeter and area (Ff for a circle = 1, other shapes < 1) were calculated using the ‘Calculate morphological properties’ function, for each nucleus. Populations based on dimension were included and excluded from further analysis by the ‘Select population’ function. Populations of nuclei smaller than 60 µm^2^ were designated as pyknotic. Populations of nuclei with roundness of under 0.65 Ff were designated as fragmented or blebbing. Neurons with nuclei that were classed as pyknotic or fragmented/blebbing were considered dead and those that were not were considered healthy. Healthy neuron nuclei were then set as the origin of neurites allowing the assignment of neurite trees to specific cells; MAP2-positive neurites were traced commencing from nuclei to their extremities. Total neurite length and average root density (nodes between neurites and nuclei) were quantified for each cell. Neurite trees were also split into segments. The branch level of each segment was scored and averaged per image. Neurite fragmentation was estimated from images by first identifying MAP2-positive neurites using the ‘Find image region’ function. This region was then split into distinct objects. The area of each MAP2-object was calculated in µm^2^, using the ‘Calculate morphology properties’ function. Fragments were selected using the ‘Select population’ function from the total object population if their area was less than or equal to 20 µm^2^. All objects over 20 µm^2^ were considered healthy neurites. The neurite fragmentation assay readout was the total number of fragments per image divided by the total number of neurons in the same image. Spinophilin-positive dendritic spines were counted on and surrounding healthy neurites, using the ‘Find image region’ and ‘Find surrounding region’ functions in the spinophilin channel. Dendritic spines were then identified and segmented using the ‘Find spots: method A’ function, solely within the two identified regions. The dendritic spine density assay readout was calculated as the number of spines per µm of healthy neurite.

### Statistical analysis

For infectivity curing experiments non-linear regression was used to calculate the effective curing concentration of ICSM18. To determine differences in control uninfected and RML prion-infected brain homogenate toxicity, non-linear regression was performed before an extra sum-of-squares *F* test. For comparisons of toxicity in cells with different *Prnp* genotypes, LogEC_50_ values were compared with unpaired two-tailed *t* tests. To determine the potential toxicity of antibodies, one-way ANOVA with Dunnett’s multiple comparisons was used to compare all antibody treatments to the no-antibody negative control. To determine the effects of antibodies on RML brain homogenate toxicity, non-linear regression was used, assuming antibody-negative RML treatment group as 0.05 nM. If data was normalised, the method for normalisation is described in figure legends. No outliers were found or removed. Sample number, statistical test, error bar type and P-value reporting level are stated in figure legends. All statistical analyses were performed in GraphPad Prism 7.

## Supplementary Information

Supplementary Figures.

## Data Availability

All data are available in the main text or the supplementary materials. ICSM18 antibody used in this study was supplied by D-Gen Limited, which is a for-profit company.

## References

[1] Collinge J (2016). Mammalian prions and their wider relevance in neurodegenerative diseases. Nature.

[2] Sandberg MK, Al-Doujaily H, Sharps B, Clarke AR, Collinge J (2011). Prion propagation and toxicity in vivo occur in two distinct mechanistic phases. Nature.

[3] Sandberg MK (2014). Prion neuropathology follows the accumulation of alternate prion protein isoforms after infective titre has peaked. Nat. Commun..

[4] Collinge J, Clarke AR (2007). A general model of prion strains and their pathogenicity. Science.

[5] Hill AF, Collinge J (2002). Species-barrier-independent prion replication in apparently resistant species. Proc. Natl. Acad. Sci..

[6] Hill AF, Collinge J (2003). Subclinical prion infection in humans and animals. Br. Med. Bull..

[7] Benilova I (2020). Highly infectious prions are not directly neurotoxic. Proc. Natl. Acad. Sci. U.S.A..

[8] Jucker M, Walker LC (2018). Propagation and spread of pathogenic protein assemblies in neurodegenerative diseases. Nat. Neurosci..

[9] Soto C, Pritzkow S (2018). Protein misfolding, aggregation, and conformational strains in neurodegenerative diseases. Nat. Neurosci..

[10] Sailer A, Büeler H, Fischer M, Aguzzi A, Weissmann C (1994). No propagation of prions in mice devoid of PrP. Cell.

[11] Manson JC (1994). 129/Ola mice carrying a null mutation in PrP that abolishes mRNA production are developmentally normal. Mol. Neurobiol..

[12] Büeler H (1993). Mice devoid of PrP are resistant to scrapie. Cell.

[13] Purro SA, Nicoll AJ, Collinge J (2018). Prion protein as a toxic acceptor of amyloid-β oligomers. Biol. Psychiatry.

[14] Corbett GT (2020). PrP is a central player in toxicity mediated by soluble aggregates of neurodegeneration-causing proteins. Acta Neuropathol..

[15] Mallucci G (2003). Depleting neuronal PrP in prion infection prevents disease and reverses spongiosis. Science.

[16] Mallucci GR (2007). Targeting cellular prion protein reverses early cognitive deficits and neurophysiological dysfunction in prion-infected mice. Neuron.

[17] Fang C, Imberdis T, Garza MC, Wille H, Harris DA (2016). A neuronal culture system to detect prion synaptotoxicity. PLoS Pathog..

[18] Solforosi L (2004). Cross-linking cellular prion protein triggers neuronal apoptosis in vivo. Science.

[19] Klöhn PC (2012). PrP antibodies do not trigger mouse hippocampal neuron apoptosis. Science.

[20] Reimann RR (2016). Differential toxicity of antibodies to the prion protein. PLoS Pathog..

[21] Wu B (2017). The N-terminus of the prion protein is a toxic effector regulated by the C-terminus. Elife.

[22] Antonyuk SV (2009). Crystal structure of human prion protein bound to a therapeutic antibody. Proc. Natl. Acad. Sci..

[23] Enari M, Flechsig E, Weissmann C (2001). Scrapie prion protein accumulation by scrapie-infected neuroblastoma cells abrogated by exposure to a prion protein antibody. Proc. Natl. Acad. Sci..

[24] Peretz D (2001). Antibodies inhibit prion propagation and clear cell cultures of prion infectivity. Nature.

[25] White AR (2003). Monoclonal antibodies inhibit prion replication and delay the development of prion disease. Nature.

[26] Klyubin I (2014). Peripheral administration of a humanized anti-PrP antibody blocks Alzheimer’s disease Aβ synaptotoxicity. J. Neurosci..

[27] Allen PB, Ouimet CC, Greengard P (2002). Spinophilin, a novel protein phosphatase 1 binding protein localized to dendritic spines. Proc. Natl. Acad. Sci..

[28] Feng J (2000). Spinophilin regulates the formation and function of dendritic spines. Proc. Natl. Acad. Sci. U.S.A..

[29] Falsig J, Aguzzi A (2008). The prion organotypic slice culture assay-POSCA. Nat. Protoc..

[30] Falsig J (2012). Prion pathogenesis is faithfully reproduced in cerebellar organotypic slice cultures. PLoS Pathog..

[31] Purro SA, Mead S, Khalili-Shirazi A, Nicoll AJ, Collinge J (2018). Reply to: Intrinsic toxicity of antibodies to the globular domain of the prion protein. Biol. Psychiatry.

[32] Song CH (2008). Effect of intraventricular infusion of anti-prion protein monoclonal antibodies on disease progression in prion-infected mice. J. Gen. Virol..

[33] Wenborn A (2015). A novel and rapid method for obtaining high titre intact prion strains from mammalian brain. Sci. Rep..

[34] Büeler H (1992). Normal development and behaviour of mice lacking the neuronal cell-surface PrP protein. Nature.

