# Supplementary material for: A high-content neuron imaging assay demonstrates inhibition of prion disease-associated neurotoxicity by an anti-prion protein antibody
Source: Sci Rep. 2022 Jun 9;12:9493. doi: 10.1038/s41598-022-13455-z (PMC9184462; doi:10.1038/s41598-022-13455-z)
Supplement: Supplementary file 1 — Supplementary Figures. [file 41598_2022_13455_MOESM1_ESM.pdf]

Supplementary Materials

Title: A high-content neuron imaging assay demonstrates inhibition of prion disease-associated neurotoxicity by an anti-prion protein antibody

Authors: Madeleine Reilly[1], Iryna Benilova[1], Azadeh Khalili-Shirazi[1], Christian Schmidt[1], Parvin Ahmed[1], Daniel Yip[1], Parmjit S. Jat[1], John Collinge[1]*

Affiliations:

[1]MRC Prion Unit at UCL, UCL Institute of Prion Diseases, University College London, Courtauld Building, 33 Cleveland Street, London W1W 7FF, United Kindgom.

*Corresponding author. Email: jc@prion.ucl.ac.uk

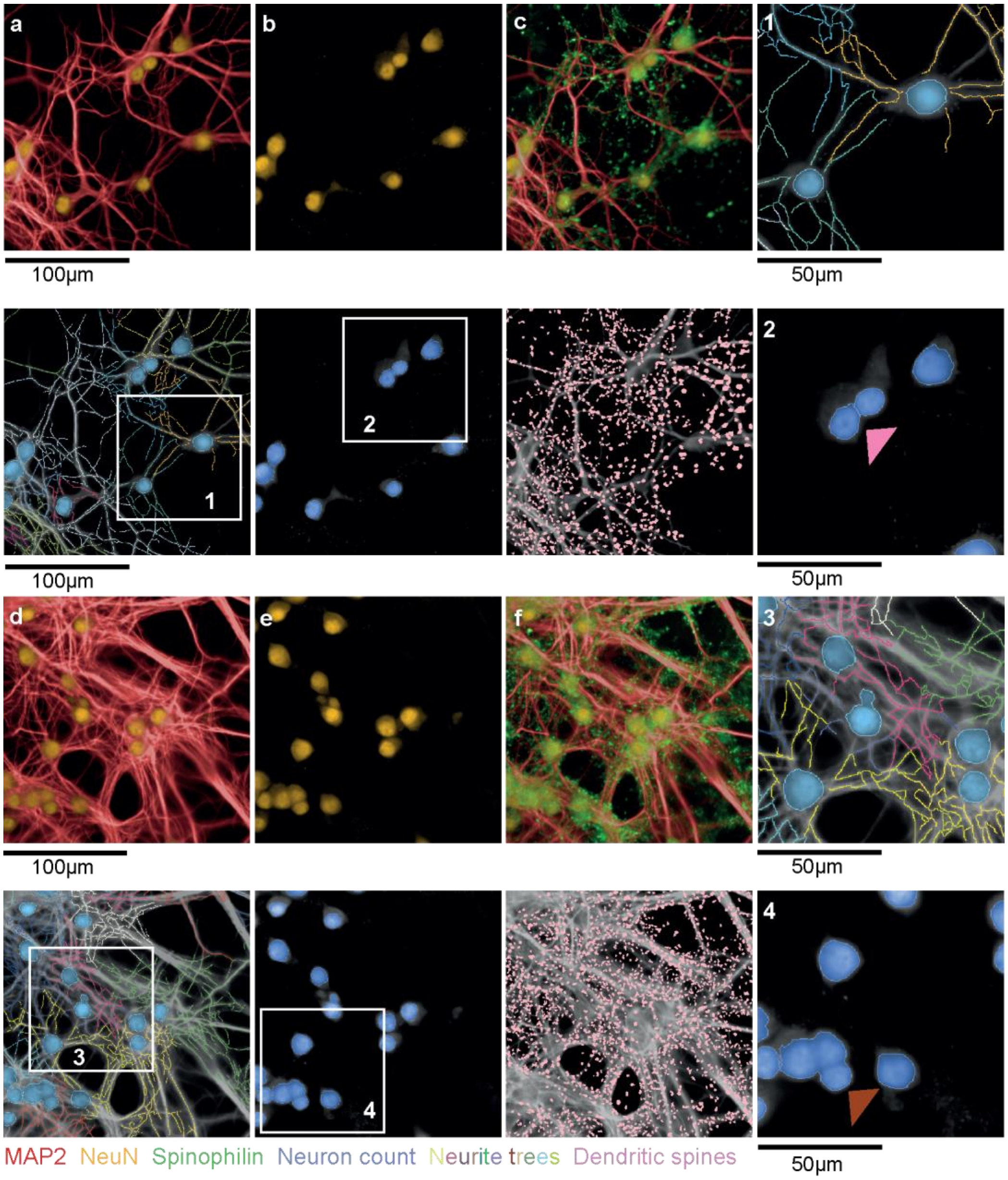

Figure S1. Optimisation of cell culture density for image analysis. Hippocampal embryonic neurons were plated at 300 (**a-c**) or 600 (**d-f**) cells/mm^2 and at 10 days old, fixed and stained against MAP2 (red), NeuN (orange) and spinophilin (green). The top panels of **a** and **d** show cells stained with MAP2 and NeuN, **b** and **e** show the NeuN channel only and **c** and **f**, all three stains. The lower panels of **a** and **d** show Columbus image analysis of neurite detection, **b** and **e**, neuron

count and **c** and **f**, dendritic spine detection. The white boxes in **a-b** and **d-e** correspond to the magnified views on the right labelled **1-4**, respectively.

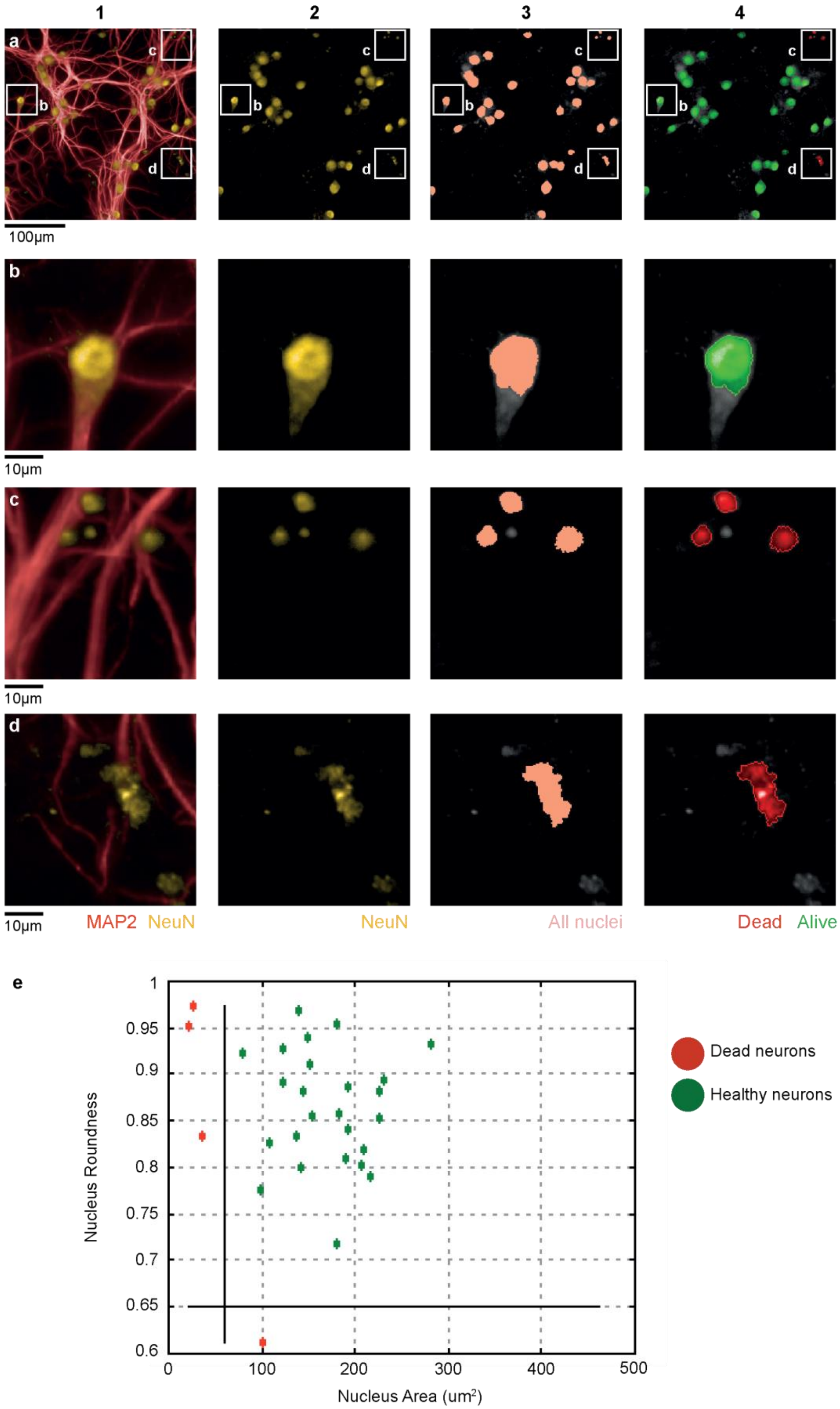

1
2
3
4
a
b
c
d
100µm
10µm
MAP2 NeuN
NeuN
All nuclei
Dead Alive
e
Nucleus Roundness
Nucleus Area (um2)
Dead neurons
Healthy neurons

Figure S2. Automatic counting of healthy neurons. (**a**) From a multichannel image (panel 1), the NeuN channel (panel 2) was selected. All NeuN-positive nuclei were firstly masked (panel 3; light pink mask) and the area and roundness of each individual nucleus was calculated. A population of healthy (green) and dead (red) nuclei were selected from total nuclei (panel 4). (**b**) A representative healthy nucleus. (**c**) A cluster of pyknotic (a condensed nuclear phenotype that is a hallmark of apoptosis) nuclei were firstly masked amongst the whole population of nuclei present. Pyknotic nuclei were selected from the whole population when their area fell under 60μm^2. (**d**) A representative fragmented nucleus was firstly masked amongst the whole population of nuclei present in the image. The fragmented nucleus was selected from the whole population as its roundness coefficient fell under 0.65 Ff: healthy nuclei are typically round. The white boxes in a relate to the magnified views in **b-d.** (**e**) Columbus software enabled a fast visualization of the dimension of all nuclear objects within the total population of one image as a scatterplot of nuclear object roundness over area. Pyknotic nuclei from **c** and the fragmented nucleus from **d** fell below the cut offs stated in the analysis script.

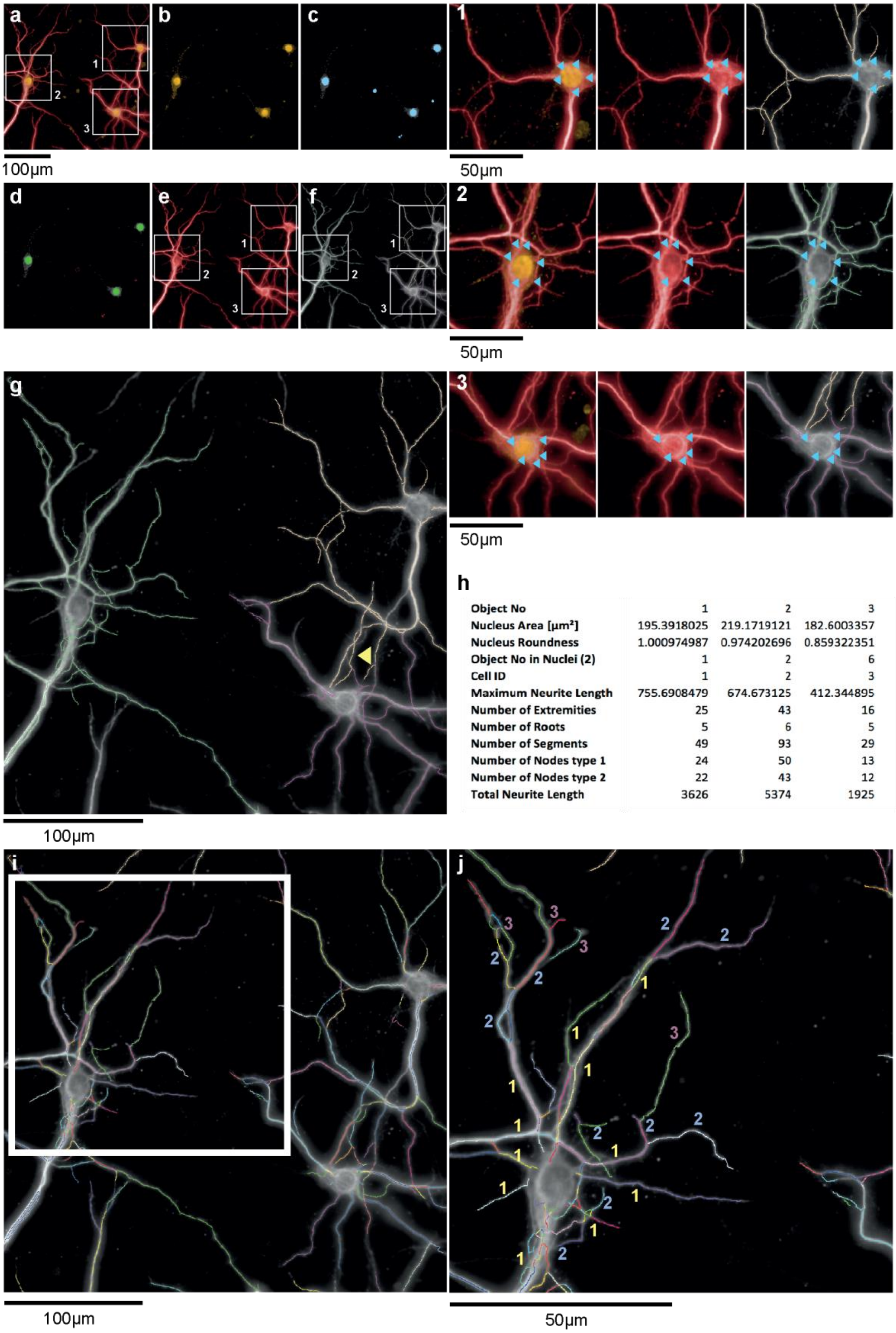

Object No	1	2	3
Nucleus Area [µm²]	195.3918025	219.1719121	182.6003357
Nucleus Roundness	1.000974987	0.974202696	0.859322351
Object No in Nuclei (2)	1	2	6
Cell ID	1	2	3
Maximum Neurite Length	755.6908479	674.673125	412.344895
Number of Extremities	25	43	16
Number of Roots	5	6	5
Number of Segments	49	93	29
Number of Nodes type 1	24	50	13
Number of Nodes type 2	22	43	12
Total Neurite Length	3626	5374	1925

Figure S3. Automatic analysis of neurite tree measurements. (**a**) Representative image of neurons prior to automatic neurite tracing; white boxes (1-3) correspond to magnified views 1-3. (**b**) Neuron nuclei were identified. (**c**) Neuron nuclei were segmented (blue) in order to measure the size and shape of each individual nucleus. (**d**) Neuron nuclei were then either classified as dead (red) or healthy (green). (**e**) MAP2-positive neurites. (**f**) Neurites were traced from only healthy nuclei, which allow the assignment of neurite trees to individual cells; white boxes labelled 1-3 correspond to magnified views 1-3 on the right (blue arrows in panels 1-3 indicate neurite roots, the number of neurites that sprout from each nucleus). (**g**) A magnification of **f** to illustrate the assignment of individual neurite trees, which are coloured distinctly, to each neuron present; yellow arrow indicates an area of neurite overlap from neurites of two different neurons; the analysis algorithm is able to assign neurite identity to the correct neuron. (**h**) A table showing the profiles of each neuron present in G in terms of neurite features; object number relates directly to cells 1-3. (**i**) The same original image but now neurite segments are individually traced where the identity of each segment is visualised by a distinct colour. (**j**) Each segment was automatically given a branch level score. The branch level analysis averages the branch level of all segments within an image. white box in **i** corresponds to magnified view in **j**.

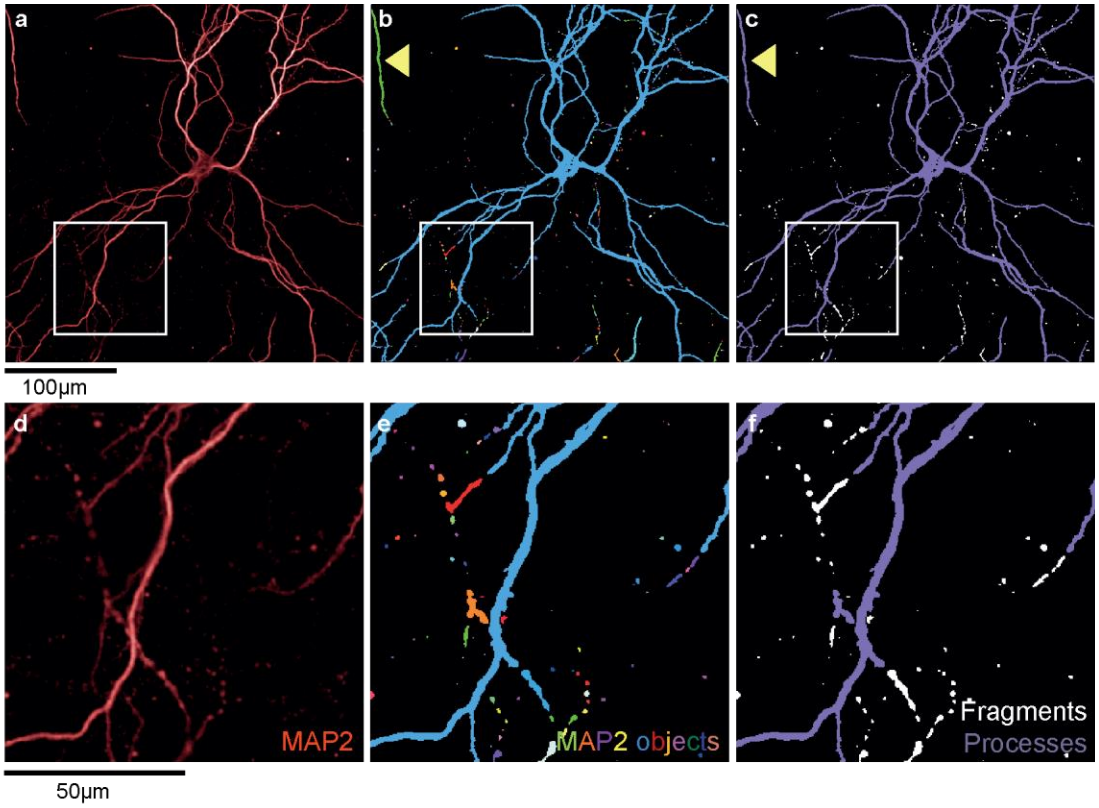

Figure S4. Automatic counting of neurite fragmentation. (**a**) Neurites were visualised by staining against MAP2. (**b**) The MAP2 image region was identified and split into objects and assigned to a spectrum of colours at random to easily visualise distinct objects; yellow arrow indicates a long expanse of healthy dendrite not connected to the main neuron. (**c**) A measurement of the area of each MAP2-positive object was taken. Objects that fell under 20μm^2 were considered fragments (white) and counted, whereas objects over this threshold were not counted as fragments (purple). Neurite fragmentation output was the number of fragments averaged over the number of neurons present in each image; yellow arrow indicates a long expanse of healthy dendrite not connected to the main neuron and not counted as a fragment. (**d-f**) Magnified views from boxes in **a-c** respectively depicting the precision of fragment detection.

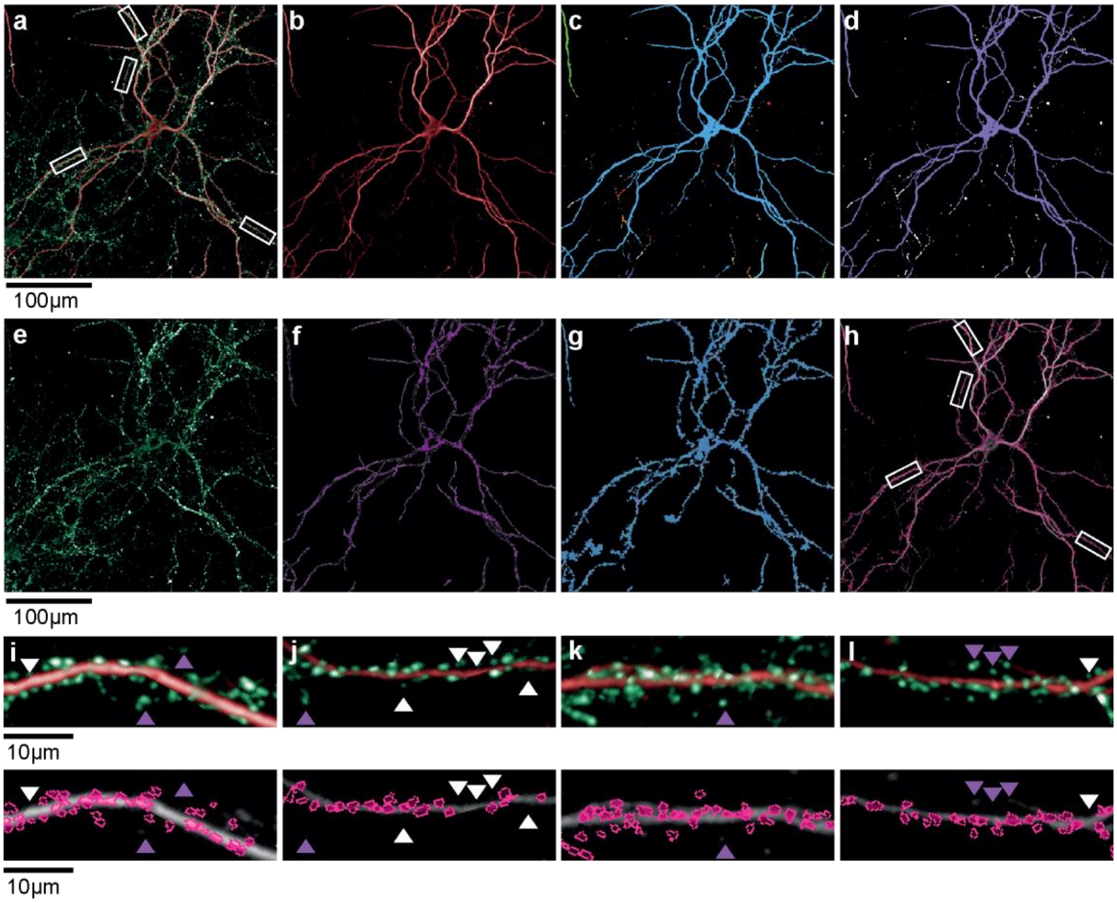

Figure S5. Automatic counting of spinophilin-positive dendritic spines**.** (**a**) Neurites were visualised by staining against MAP2 and dendritic spines marked by spinophilin. (**b**) The MAP2 image region was identified. (**c**) The MAP2 image region was split into objects. The area of each object was measured. Objects that fell under 20µm^2 were considered as fragments (white) and removed from the region. (**d**) Spines were quantified from only the remaining region of healthy neurites. (**e**) Spinophilin stain. (**f-g**) Spine counts were made by segmenting a region of spinophilin solely within **f** and surrounding **g**, the healthy MAP2 dendritic region of d. (**h**) Spinophilin-positive spots, considered dendritic spines, were then counted from within the perimeter of **g** that surrounded the identified region in **f**, which was approximately 0-4µm adjacent to healthy processes. (**i-l**) Magnified views of four white rectangles from the raw image in **a** (top) and the analysis image in **h** (bottom) of 40µm expanses of dendrite depicting the accuracy of dendritic

spine counting; white arrows relate to dull spinophilin-positive spots that were not counted as spines; purple arrows relate to spinophilin-positive spots that did not sit within the identified perimeter surrounding dendrites to be considered spines.

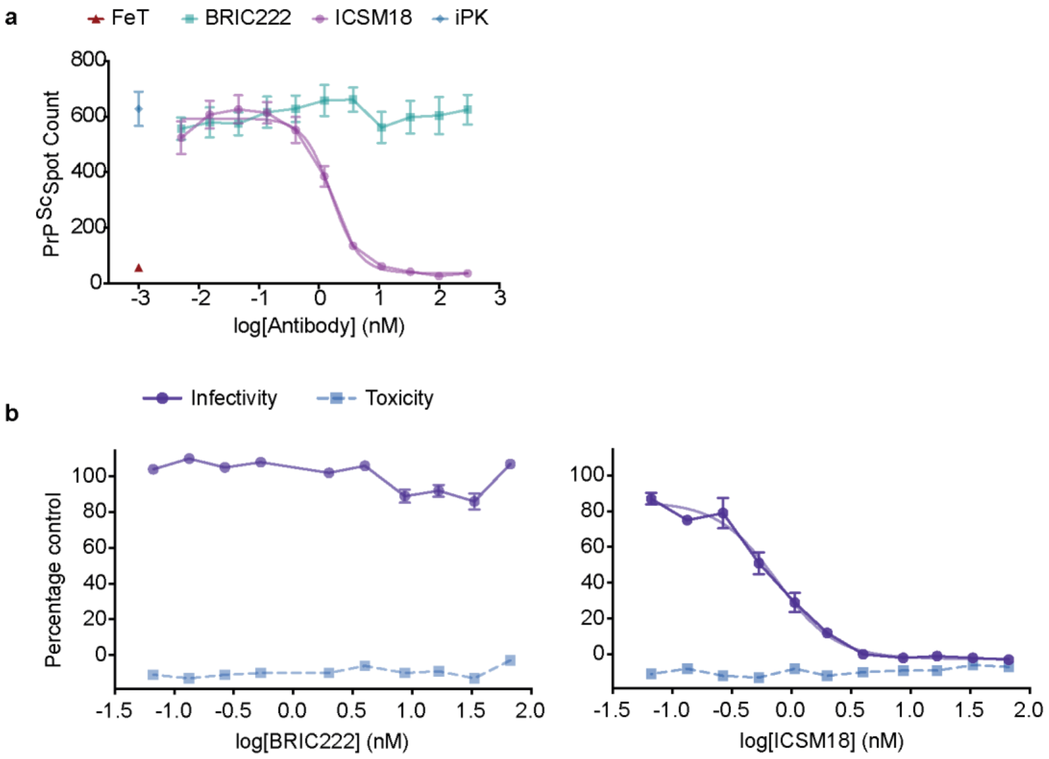

Figure S6. ICSM18 cures RML prion-infected cells and binds tightly to PrP^{C} whilst remaining non-cytotoxic. (**a**) Chronically RML prion-infected iPK1 cells were incubated for 4 days with varying concentrations of antibody. The curing of infectivity was assessed by clearance of PrP^{Sc} as quantified by PrP ELISPOT. ICSM18 (pink) cured iPK1 cells, in contrast to the control antibody, BRIC222 (green), which did not. FeT (red) was used as a positive control to effectively cure infection. iPK cells without antibody were used as a negative control. Results are the mean±SEM of *n=6* independent tests; non-linear regression computed the ICSM18 inhibitory curing concentration with Y=Bottom + (Top-Bottom)/(1+10^(($LogEC_{50}$-X)*HillSlope)). (**b**) Chronically RML prion-infected iPK-1 cells were incubated with serial dilutions of BRIC222 (left) and ICSM18 (right) for 4 days before viability assay (light blue) and PrP^{Sc} detection (purple); Results are normalised as a percentage of control and shown as mean±SEM of one of three representative experiments; non-linear regression computed the ICSM18 inhibitory curing concentration with Y=Bottom + (Top-Bottom)/(1+10^(($LogEC_{50}$-X)*HillSlope)).
